# The pungent substances piperine, capsaicin, 6-gingerol and polygodial inhibit the human two-pore domain potassium channels TASK-1, TASK-3 and TRESK

**DOI:** 10.3389/fphar.2013.00141

**Published:** 2013-11-18

**Authors:** Leopoldo R. Beltrán, Corinna Dawid, Madeline Beltrán, Guenter Gisselmann, Katharina Degenhardt, Klaus Mathie, Thomas Hofmann, Hanns Hatt

**Affiliations:** ^1^Department of Cell Physiology, Ruhr-University-BochumBochum, Germany; ^2^Chair for Food Chemistry and Molecular Sensory Science, Technische Universität MünchenFreising, Germany

**Keywords:** K_2P_ channels, pungency, piperine, capsaicin, 6-gingerol, polygodial

## Abstract

For a long time, the focus of trigeminal chemoperception has rested almost exclusively on TRP channels. However, two-pore domain (K_2P_) potassium channels have recently been identified as targets for substances associated with typical trigeminal sensations, such as numbing and tingling. In addition, they have been shown to be modulated by several TRP agonists. We investigated whether the pungent substances piperine, capsaicin, 6-gingerol and polygodial have an effect on human K_2P_ channels. For this purpose, we evaluated the effects of these pungent substances on both wild-type and mutant K_2P_ channels by means of two-electrode voltage-clamp experiments using *Xenopus laevis* oocytes. All four pungent substances were found to inhibit the basal activity of TASK-1 (K_2P_ 3.1), TASK-3 (K_2P_ 9.1), and TRESK (K_2P_ 18.1) channels. This inhibitory effect was dose-dependent and, with the exception of polygodial on TASK-1, fully reversible. However, only piperine exhibited an IC_50_ similar to its reported EC_50_ on TRP channels. Finally, we observed for TASK-3 that mutating H98 to E markedly decreased the inhibition induced by piperine, capsaicin, and 6-gingerol, but not by polygodial. Our data contribute to the relatively sparse knowledge concerning the pharmacology of K_2P_ channels and also raise the question of whether K_2P_ channels could be involved in the pungency perception of piperine.

## INTRODUCTION

In modern cuisine, numerous spices, such as black peppercorns (*Piper nigrum*), chili pepper (*Capsicum annuum*), mountain pepper (*Tasmannia lanceolata*) or ginger (*Zingiber officinalis*), have been appreciated worldwide for their ability to add a pungent orosensory impression to dishes. Today, after several genetic and functional studies, the pungency of these spices is believed to be based mainly on the activation of two members of the transient receptor potential (TRP) family, TRPV1 and TRPA1. These two polymodic, non-selective cation channels, which also play a role in pain perception, are expressed on free afferent nerve endings of trigeminal neurons in the oral cavity (Calixto et al., 2005).

Over the years, a series of non-volatile phytochemicals, which were able to induce a pungent sensation but varied widely in molecular structure, have been reported to activate these two ion channels. For example, capsaicin, one of the most pungent phytochemicals from chili peppers, was the first agonist reported for TRPV1 (Caterina et al., 1997). Piperine, one of the key molecules responsible for the pungency of black pepper (Dawid et al., 2012), has also been shown to activate TRPV1 (McNamara et al., 2005). Similarly, other pungent *Piper nigrum* amides such as, e.g., piperolein B, were reported to activate the ion channels TRPA1 and TRPV1 (Correa et al., 2010; Okumura et al., 2010). In the same way, polygodial, the pungent drimane-type sesquiterpene dialdehyde from mountain pepper (Barnes and Loder, 1962), and vanilloids such as, e.g., 6-gingerol from ginger rhizomes (Belitz et al., 2004), have been identified as TRPA1 and TRPV1 activators (Dedov et al., 2002; Witte et al., 2002; Bandell et al., 2004; Calixto et al., 2005; André et al., 2006; Escalera et al., 2008; Iwasaki et al., 2009; Morera et al., 2012).

A vast number of other pungent materials also activate members of the TRP family, e.g., alicine from garlic, eugenol from clove, cinnamaldehyde from cinnamon, and extracts of habanero pepper, Thai green chili or mustard oil (Caterina et al., 1997; Bandell et al., 2004; Calixto et al., 2005). Although the role of TRP channels in chemoperception is well accepted, there are some facts suggesting other possible targets for some of these pungent substances. For example, piperine and capsaicin have been shown to present a stimulus-induced recovery in human psychophysical tests, in which volunteer subjects have reported that, after an initial desensitization period, under recurrent capsaicin or piperine stimulation, the sensation of irritation grew towards un-desensitized levels (Green, 1998). This is in contradiction to TRPV1 responses in heterologous systems (Caterina et al., 1997). Moreover, human beings were found to be able to distinguish between 33 and 330 μM of capsaicin, although these concentrations are much higher than those needed for TRPV1 saturation (Green, 1996).

**FIGURE 1:**
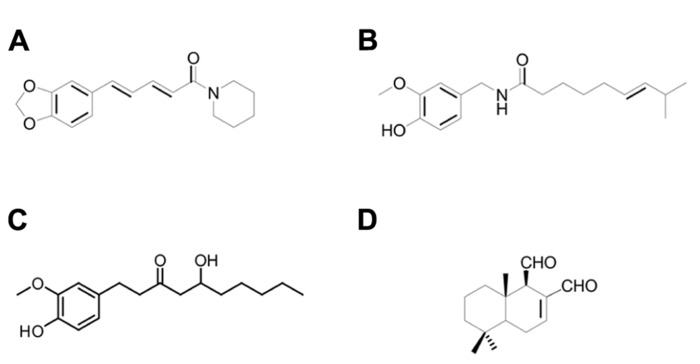
**Chemical structure of (A) piperine, (B) capsaicin, (C) 6-gingerol and (D) polygodial**.

Two-pore domain (K_2P_, KCNK) channels are plausible candidates for a complementary role in the chemoperception of these pungent substances. They are potassium-selective channels that tend to be constitutively open and are thought to be responsible for the leak potassium current present in every excitable cell. K_2P_ channels allow a constant efflux of K^+^, which helps to maintain the cell at hyperpolarized potentials and may even counterbalance excitatory stimuli (Goldstein et al., 2001; Enyedi and Czirják, 2010). Recently, in rat trigeminal neurons, three members of this family, namely rTREK-1 (K_2P_2.1, KNCK2), rTREK-2 (K_2P_10, KCNK10) and rTRAAK (K_2P_4, KCNK4), have been shown to co-express with TRP channels in the same neurons (Yamamoto et al., 2009). In mice, mTREK-1 (K_2P_2, KCNK2) and mTRAAK (K_2P_4, KCNK4) have been reported to cooperate with TRP channels for the appropriate perception of ambient temperature, meaning that mice lacking both TREK-1 and TRAAK exhibited heat and cold hyperalgesia (Noël et al., 2009). Furthermore, we have recently shown that human TREK-1, TRAAK, and TREK-2 are activated by 2-APB, a blocker but also a common activator for several TRP channels (Beltrán et al., 2013), adding this compound to the list of TRP agonists/antagonists that have an effect on K_2P_ channels, e.g., pH, temperature, anandaminde, etc. (Enyedi and Czirják, 2010). In addition to these facts, three K_2P_ channels, namely mTASK-1 (K_2P_3, KCNK3), mTASK-3 (K_2P_9, KCNK9) and mTRESK (K_2P_18, KCNK 18), have been identified in mice as the molecular targets of hydroxy-α-sanshool, a substance that induces typical trigeminal sensations, such as numbing and tingling (Bautista et al., 2008). Despite all these findings, there have been almost no further studies on the role that KCNK channels may play as receptors for other substances affecting the trigeminal system and, although they are now regarded as key players in processes ranging from apoptosis and oxygen chemoperception to depression (Enyedi and Czirják, 2010), little is known about their pharmacology. Therefore, we studied the effects of four dietary pungent substances, namely capsaicin, 6-gingerol, piperine and polygodial (**Figure 1**), on the human orthologues of the six above mentioned K_2P_ channels and found that piperine inhibited human TASK-1, TASK-3 and TRESK in a dose-dependent manner, exhibiting an IC_50_ for TASK-1 similar to its reported EC_50_ on TRPV1. The other 3 substances were also able to induce a dose-dependent inhibition on TASK-1, TASK-3 and TRESK, but only at concentrations relatively higher than their reported EC_50_s on TRP channels. Interestingly, we also observed that for TASK-3, mutating H98 to E markedly decreases the inhibition induced by piperine, capsaicin and 6-gingerol.

## METHODS

### MOLECULAR BIOLOGY

Human TASK-1 (K_2P_3.1, KCNK3) cloned into the expression vector pIRES-CD8 was a gift from Dr. Fabrice Duprat (Institut National de la Santé et de la Recherche Médicale, Antipolis, France), and hTRESK (K_2P_18.1, KCNK18), cloned into the plasmid pEXO, was a gift from Prof. Dr. P. Enyedi (Semmelweis University, Budapest, Hungary). These plasmids were linearized with the restriction enzymes *Bam*HI and *Xba*I, respectively, and then used as templates for *in vitro* transcription. hTASK-3 (K_2P_9.1, KCNK9) cloned into the expression vector pBR4-TOPO, hTRAAK (K_2P_4.1, KCNK4) cloned into pBluescript SK+, hTREK-2 (K_2P_10.3, a slightly longer isoform of KCNK10 with a distinct *N*-terminus compared to isoform 1; Staudacher et al., 2011) cloned into pBR4-TOPO and hTREK-1 (K_2P_2, KCNK2) cloned into pDNR-dual were purchased from Imagenes (Berlin, Germany). hTREK-1 and hTREK-2 cDNA inserts were later subcloned into pSGEM. The plasmids were linearized with the restriction enzymes *Ssp*I for pBR4-TOPO, *Not*I for pBluescript SK+ and *Pac*I for hTREK-1 and hTREK-2 subcloned into pSGEM, to generate a linear cDNA template for *in vitro* transcription.

Capped RNAs were synthesized in the presence of the capping analog m7G(5′)ppp(5′)G using the AmpliCap-T7 Message Maker kit (Epicentre, Madison, USA). cRNA was dissolved in nuclease-free water to give a final concentration of 100 ng/μL. Mutants or chimeras for hTASK-3 were constructed by standard overlap extension PCR methods and cloned into the pCDNA3 vector.

### *Xenopus laevis* OOCYTES PREPARATION

The expression in *Xenopus laevis* oocytes was performed essentially as described by Vogt-Eisele et al. (2007). Briefly, mature female *Xenopus laevis* frogs were anesthetized with MS-222 at 0.15%, and surgery was performed according to standard methods. The extracted ovarian lobes were placed into Ca^2^^+^-free Barth’s solution containing collagenase type II (Worthington Biochemical Corporation, Lakewood, NJ, USA) and kept for 90 min on a shaker at 40 rpm (room temperature). After that time, healthy stage V and VI oocytes were selected for injection with cRNA coding for the proteins of interest. For each channel, 30 nL of cRNA (100 ng/μL) was injected using a Microinjector Micro 4 TM (World Precision Instruments, Sarasota, Florida, USA). After injection, the oocytes were kept in ND96 (96 mM NaCl; 2 mM KCl; 1.8 mM CaCl_2_; 1 mM MgCl_2_; 2.5 mM sodium pyruvate 3 mM; HEPES 10 mM; pH 7.2), and measurements were performed 24 to 72 h after injection.

The oocytes were placed in a 100 μL chamber that was perfused with frog Ringer’s solution (115 mM NaCl; 2.5 mM KCl; 1.8 mM CaCl_2_ and 10 mM HEPES pH 7.2). Chemicals to be evaluated were dissolved in this solution before being manually applied to the oocytes using an automatic pipette (research pro, Eppendorf, Germany). The application time was usually three ramps (approximately 10 s). For substances that did not show a complete recovery, only one concentration per oocyte was evaluated. For recording, a Turbo Tec-03x (npi electronic GmbH, Tamm, Germany) amplifier was used, with an acquisition rate of 1000 Hz and a low pass filter of 20 Hz. Borosilicate glass capillaries were pulled with a Kopf vertical pipette puller. The pulled glass capillaries were filled with 3 M KCl.

### TWO-ELECTRODE VOLTAGE CLAMP MEASUREMENTS

A ramp series protocol was used to evaluate the activity of the channels. The voltage ramps consisted of a starting constant of -100 mV for 300 ms, followed by a ramp to +50 mV in 700 ms, then a constant at +50 mV for 150 ms, and a final constant at -40 mV for 500 ms. The time interval between the ramps was 2 s. To evaluate the effect of an applied substance, we averaged the current registered at the final 50 ms of the +50 mV constant in the three ramps showing the maximal response to a substance, and divided that by the average obtained from three ramps prior to the application. In most cases, the currents elicited by this voltage-clamp ramp series protocol were more than ten times larger than the background currents observed in non-injected oocytes, while also presenting the described outward rectification under physiological concentrations of intracellular and extracellular K^+^(Goldstein et al., 2001; Enyedi and Czirják, 2010). Due to these characteristics, no leakage subtraction was considered necessary. Oocytes not fulfilling these characteristic were not considered for data analysis.

The data were collected using the *Cellworks Reader 3.7* software (npi instruments, Tamm, Germany) and analyzed with *Clampfit v10.2.0.14* (MDS Analytical Technologies, Sunnyvale, USA). Curve fitting was performed using the 4-parameter Hill equation (Sigmaplot, Systat software inc., San Jose, USA). IC_50_ values refer to the concentration at which 50% of the maximum observed effect was seen. The error associated with these values represents the error in the fit to the mean data at each concentration. Bar diagram data represent the mean +/- SEM. Paired *T*-test was used to evaluate the statistical significance of the results, *P* < 0.05 was considered statistically significant and is marked with one star, *P* < 0.01 is marked with two stars, and *P* < 0.001 is marked with three stars.

### DRUGS, CHEMICALS, REAGENTS AND OTHER MATERIALS (INCLUDING SOURCES)

Capsaicin, DMSO, piperine, and 6-gingerol were purchased from Sigma-Aldrich (Steinheim, Germany), while polygodial was obtained from Santa Cruz Biotechnology (Heidelberg, Germany). Stock solutions of 100 and 300 mM diluted in DMSO were made and stored at -20°C. Aliquots were then taken to obtain the desired concentrations. Piperine and 6-gingerol were purified by means of RP-HPLC in a purity of >98%.

## RESULTS

### PUNGENT COMPOUNDS INHIBIT TASK-1, TASK-3 AND TRESK IN A DOSE-DEPENDENT MANNER

#### Piperine

**FIGURE 2:**
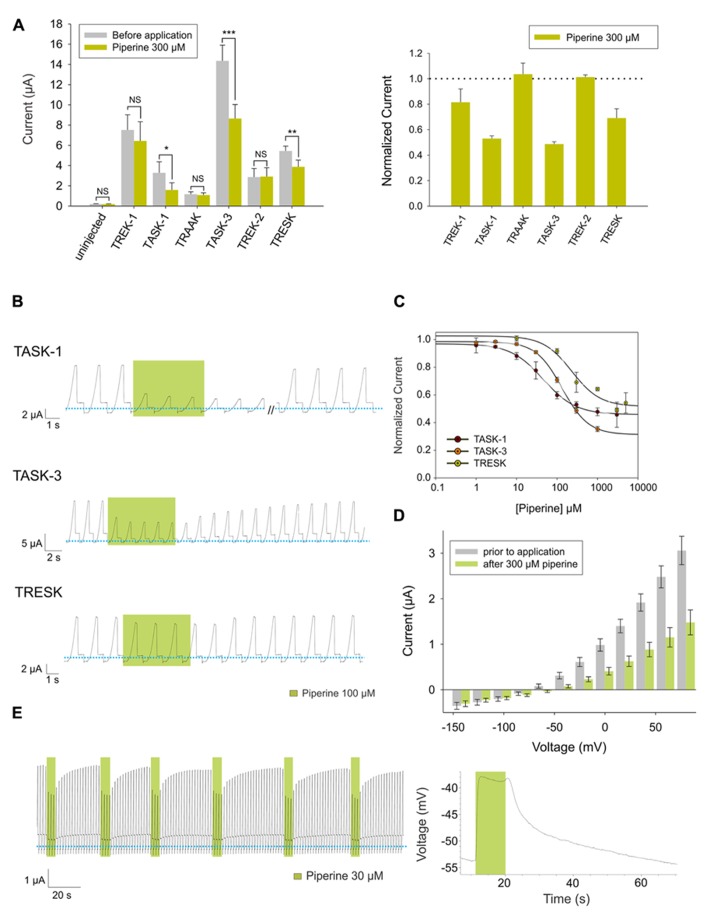
**(A)** *Left* (from left to right) absolute currents presented by: uninjected *Xenopus* oocytes, oocytes injected with cRNA coding for hTREK-1, hTASK-1, hTRAAK, hTASK-3, hTREK-2 and hTRESK, before and after the application of 300 μM piperine. The currents were registered at the final 50 ms of the +50 mV constant (see methods for a description of the ramp protocol used). Note that the amount of current carried by uninjected oocytes is negligible in comparison to oocytes expressing a K_2P_ channel. *Right*. Normalized currents showing the effect of 300 μM piperine on *Xenopus* oocytes injected with cRNA coding for hTREK-1, hTASK-1, hTREK-1, hTASK-3, hTREK-2 and hTRESK. For each group, the current was normalized to the current registered prior to the application of 300 μM piperine (dotted line). All data are expressed as the mean +/- SEM. N of 3–6 for injected oocytes, N of 10 for uninjected oocytes. *P < 0.05, **P < 0.01, ***P < 0.001. **(B)** Representative voltage clamp recordings of *Xenopus laevis* oocytes expressing hTASK-1 (top), hTASK-3 (middle) and hTRESK (bottom), before, during and after the application of 100 μM piperine. In each case, piperine induced a fully reversible inhibition; for TASK-1, however, due to the prolonged recovery time, 40 ramps were not included. Dotted line represents zero current. **(C)** Dose-response curves for piperine on hTASK-1 (red circles), hTASK-3 (orange circles) and hTRESK (green circles). The IC_50_ values are listed in the text and in **Table 1**. N of 3-6 oocytes. For each measurement, the current was normalized to the current registered prior to the application of piperine. **(D)** hTASK-1 currents recorded before (gray bars) and after (green bars) the application of 300 μM piperine. The currents were elicited by voltage pulses from -140 to +80 mV in 20-mV steps, 500 ms duration from a holding potential of -80 mV. Values were obtained during the last 50 ms of the voltage pulses. N of 6 oocytes. **(E)** *Left:* representative voltage clamp recording of a *Xenopus* oocyte expressing hTASK-1 repetitively exposed to 30 μM piperine. *Right:* current clamp recording of a *Xenopus* oocyte expressing hTASK-1 exposed to 100 μM piperine, which led to a membrane depolarization of approximately 15 mV.

To investigate whether piperine has an effect on human K_2P_ channels, we used *Xenopus laevis* oocytes as expression system for human TREK-1, TASK-1, TRAAK, TASK-3, TREK-2, and TRESK. We then evaluated whether bath-applied piperine could produce an effect on the basal current of these channels. Through this approach, we observed a significant inhibition of TASK-1, TASK-3 and TRESK upon exposure to 300 μM piperine (**Figure 2A**).

All three channels were partially and reversibly inhibited by piperine (**Figure 2B**). This inhibition was dose-dependent, and, when evaluated at +50 mV, piperine exhibited an IC_50_ value of 45 ± 3.9 μM on TASK-1, which is in the same range as reported for TRPV1 channels (McNamara et al., 2005). TASK-3 and TRESK showed IC_50_ values of 133.3 ± 5.9 μM and 230.7 ± 105 μM, respectively (**Figure 2C** and **Table 1**). The current-voltage relationship showed the inhibitory effect of piperine to be more pronounced at potentials more positive than the potassium reversal potential (**Figure 2D** for TASK-1, data for TASK-3 and TRESK not shown). This is most likely due to the fact that K_2P_ channels behave as outward rectifiers under the given ionic conditions, and the low amount of inward current that they carry is not entirely responsible for the leakage current presented at potentials more negative than the K_rev_ (included because no leak substraction was performed). In psychophysical tests, exposure to piperine is reported to lead to desensitization (Green, 1996). This desensitization, however, can be reversed by recurrent stimulation, a phenomenon known as stimulus-induced recovery which does not match the behavior of heterologously expressed TRPV1 when exposed to piperine (McNamara et al., 2005). We therefore assessed the effect of repetitive piperine application on TASK-1, the channel in which it presented its highest potency, but failed to observe any marked difference in the response of TASK-1 to repetitive applications of 30 μM piperine (**Figure 2E**, left). Finally, to study the effect of inhibiting this K_2P_ channel at the membrane potential level, *Xenopus* oocytes expressing TASK-1 were evaluated in current-clamp mode and exposed to piperine, which led to a reversible depolarizing change in the membrane potential (**Figure 2E**, right).

**Table 1:** IC_50_ values for pungent substances on human K_2P_ channels.

	TASK-1	TASK-3	TRESK
Piperine	45 ± 3.9	133.3 ± 5.9	230.7 ± 105
Capsaicin	61.1 ± 4.0	41.8 ± 3.6	70.2 ± 5.3
6-Gingerol	227.4 ± 46.5	384.6 ± 55.6	155.0 ± 70.9
Polygodial	484.5 ± 18.2	328 ± 24.7	ND

*All values are expressed in μM. ND, not determined.*

#### Capsaicin

**FIGURE 3:**
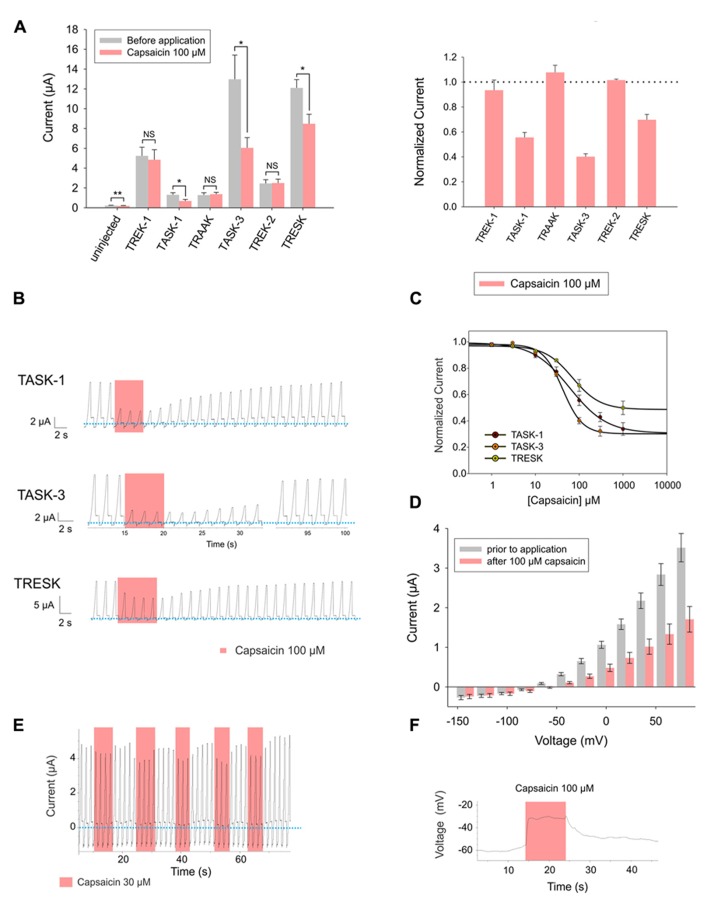
**(A)** *Left* (from left to right) absolute currents presented by: uninjected *Xenopus* oocytes, oocytes injected with cRNA coding for hTREK-1, hTASK-1, hTRAAK, hTASK-3, hTREK-2 and hTRESK, before and after the application of 100 μM capsaicin. *Right*: normalized currents showing the effect of 100 μM capsaicin on *Xenopus* oocytes injected with cRNA coding for hTREK-1, hTASK-1, hTRAAK, hTASK-3, hTREK-2 and hTRESK. For each group, the current was normalized to the current registered prior to the application of 100 μM capsaicin (dotted line). All data are expressed as the mean +/- SEM. N of 3–6 oocytes. *P < 0.05, **P < 0.01. **(B)** Representative voltage clamp recordings of *Xenopus laevis* oocytes expressing TASK-1 (top), TASK-3 (middle) and TRESK (bottom), before, during and after the application of 100 μM capsaicin, showing in every case a fully reversible inhibition. Dotted line represents zero current. **(C)** Dose-response curves for capsaicin on TASK-1 (red circles), TASK-3 (orange circles) and TRESK (green circles). The IC_50_ values are listed in the text and in **Table 1**. N of 3–6 oocytes. For each measurement, the current was normalized to the current registered prior to the application of capsaicin. **(D)** hTASK-3 currents recorded before and after the application of 100 μM capsaicin. The currents were elicited by voltage pulses from -140 to +80 mV in 20 mV steps, 500 ms duration from a holding potential of -80 mV. Values were obtained during the last 50 ms of the voltage pulses. N of 6 oocytes. **(E)** Representative voltage clamp recording of a *Xenopus* oocyte expressing TASK-3 repetitively exposed to 30 μM capsaicin. **(F)** Current clamp recording of a *Xenopus* oocyte expressing TASK-3 exposed to 100 μM capsaicin, which led to a membrane depolarization of approximately 30 mV.

Following the same experimental approach as reported above for piperine, we expressed human TREK-1, TASK-1, TRAAK, TASK-3, TREK-2, and TRESK channels in *Xenopus laevis* oocytes and evaluated whether bath-application of capsaicin, the pungent principle of chili peppers, could affect the basal current of these channels. TASK-1, TASK-3, and TRESK showed significant decreases in basal activity when exposed to 100 μM capsaicin. Interestingly, non-injected oocytes were also significantly inhibited by 100 μM capsaicin; however, the magnitude of the background current presented by non-injected oocytes, both before and after application, was negligible in comparison to the current presented by injected oocytes (**Figure 3A**).

Capsaicin strongly and reversibly inhibited the high background current possessed by TASK-1, TASK-3 and TRESK-injected *Xenopus laevis* oocytes (**Figure 3B**). All three channels were inhibited by capsaicin, with IC_50_ values in approximately the same range: 41.8 ± 3.6 μM, 61.1 ± 4.0 μM and 70.2 ± 5.3 μM for TASK-3, TASK-1 and TRESK, respectively (**Figure 3C** and **Table 1**). Similarly to what we observed for piperine on TASK-1, the inhibitory effect of capsaicin upon TASK-3 was more pronounced at potentials more positive than the potassium reversal potential (**Figure 3D**) and the channel behaved similarly when exposed to repetitive applications of capsaicin (**Figure 3E**). Additionally, this inhibition of potassium efflux markedly changed the membrane voltage towards more positive potentials as could be observed in the current-clamp mode (**Figure 3F**).

#### 6-Gingerol

**FIGURE 4:**
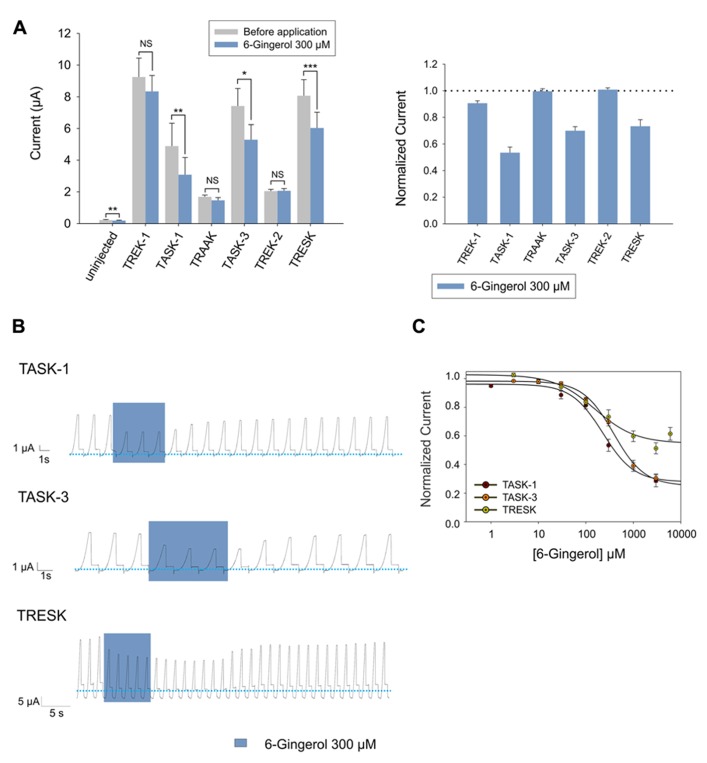
**(A)** *Left* (from left to right) absolute currents presented by: uninjected *Xenopus* oocytes, oocytes injected with cRNA coding for hTREK-1, hTASK-1, hTRAAK, hTASK-3, hTREK-2 and hTRESK, before and after the application of 300 μM 6-gingerol. *Right* normalized currents showing the effect of 300 μM 6-gingerol on *Xenopus* oocytes injected with cRNA coding for hTREK-1, hTASK-1, hTRAAK, hTASK-3, hTREK-2 and hTRESK. For each group, the current was normalized to the current registered prior to the application of 300 μM 6-gingerol (dotted line). All data are expressed as the mean +/- SEM. N of 9–18 oocytes. *P < 0.05, **P < 0.01, ***P < 0.001. **(B)** Representative voltage clamp recordings of *Xenopus laevis* oocytes expressing TASK-1 (top), TASK-3 (middle) and TRESK (bottom), before, during and after the application of 300 μM 6-gingerol, showing fully reversible inhibition in all cases. Dotted line represents zero current. **(C)** Dose-response curves for 6-gingerol on TASK-1 (red circles), TASK-3 (orange circles) and TRESK (green circles). N of 3–19 oocytes. For each measurement, the current was normalized to the current registered prior to the application of 6-gingerol.

Continuing with our screening of alimentary pungent substances, we studied the effect of the ginger component 6-gingerol and found that it induced an inhibition that was fully reversible and dose-dependent on TASK-1, TASK-3, and TRESK, respectively (**Figures 4A–C**). Interestingly, 300 μM of 6-gingerol significantly inhibited the endogenous background current presented by uninjected *Xenopus* oocytes as already observed for capsaicin. Again, the magnitude of the background current before and after the application of 300 μM 6-gingerol, was negligible when compared to the background current presented by *Xenopus* oocytes expressing any of the evaluated K_2P_ channels (**Figure 4A**). All three channels were inhibited by 6-gingerol in a dose-dependent manner, with IC_50_ values of 155.0 ± 70.9 μM, 227.4 ± 46.5 μM and 384.6 ± 55.6 μM for TRESK, TASK-1, and TASK-3 respectively (**Figure 4C** and **Table 1**).

#### Polygodial

**FIGURE 5:**
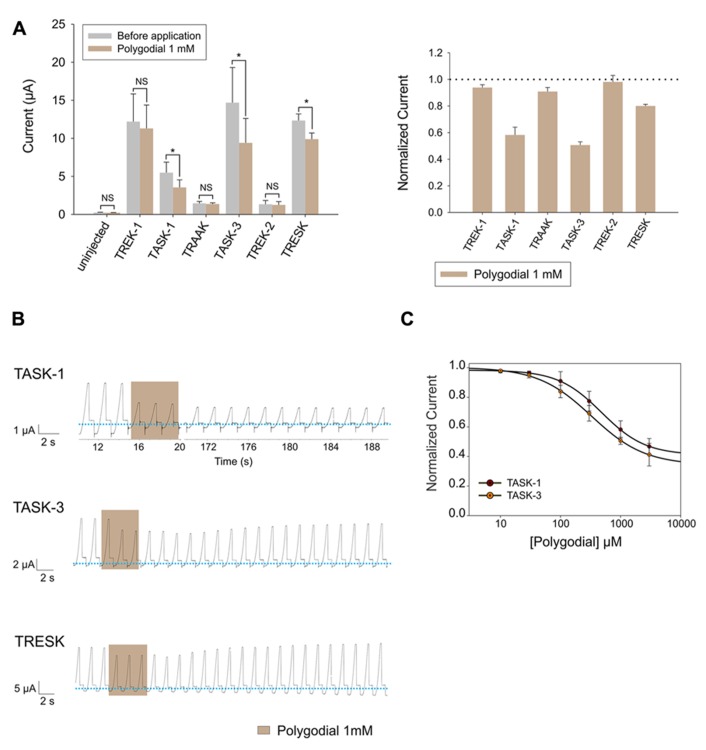
**(A)** *Left* (from left to right) absolute currents presented by: uninjected *Xenopus* oocytes, oocytes injected with cRNA coding for hTREK-1, hTASK-1, hTRAAK, hTASK-3, hTREK-2 and hTRESK, before and after the application of 1 mM polygodial. *Right*. Normalized currents showing the effect of 1 mM polygodial on *Xenopus* oocytes injected with cRNA coding for hTREK-1, hTASK-1, hTRAAK, hTASK-3, hTREK-2 and hTRESK. For each group, the current was normalized to the current registered prior to the application of 1 mM polygodial (dotted line). All data are expressed as the mean +/- SEM. N of 3 to 6 oocytes. *P < 0.05. **(B)** Representative voltage clamp recordings of *Xenopus laevis* oocytes expressing TASK-1 (top), TASK-3 (middle) and TRESK (bottom), before, during and after the application of 1 mM polygodial, showing a partially reversible inhibition for TASK-1 and TASK-3. Dotted line represents zero current. **(C)** Dose-response curves for polygodial on TASK-1 (red circles) and TASK-3 (orange circles). The IC_50_ values are listed in the text and in **Table 1**. N of 3–5 oocytes. For each measurement, the current was normalized to the current registered prior to the application of polygodial.

Lastly, we evaluated the effect of polygodial, the pungent key principle of tasmanian mountain pepper, on human K_2P_ channels. We observed that 1 mM could significantly inhibit the basal activity of TASK-1, TASK-3 and, to a lesser degree, also TRESK (**Figure 5A**). Interestingly, the polygodial-induced inhibition of TASK-1 and TASK-3 was only partially reversible, even after a prolonged washout period (**Figure 5B**). TASK-1 and TASK-3 were both inhibited by polygodial in a dose-dependent manner, with IC_50_ values of 328 ± 24.7 μM and 484.5 ± 18.2 μM for TASK-3 and TASK-1, respectively (**Figure 5C**).

#### The histidine residue at position 98 of TASK-3 is involved in the inhibitory effect induced by capsaicin, piperine and 6-gingerol

**FIGURE 6:**
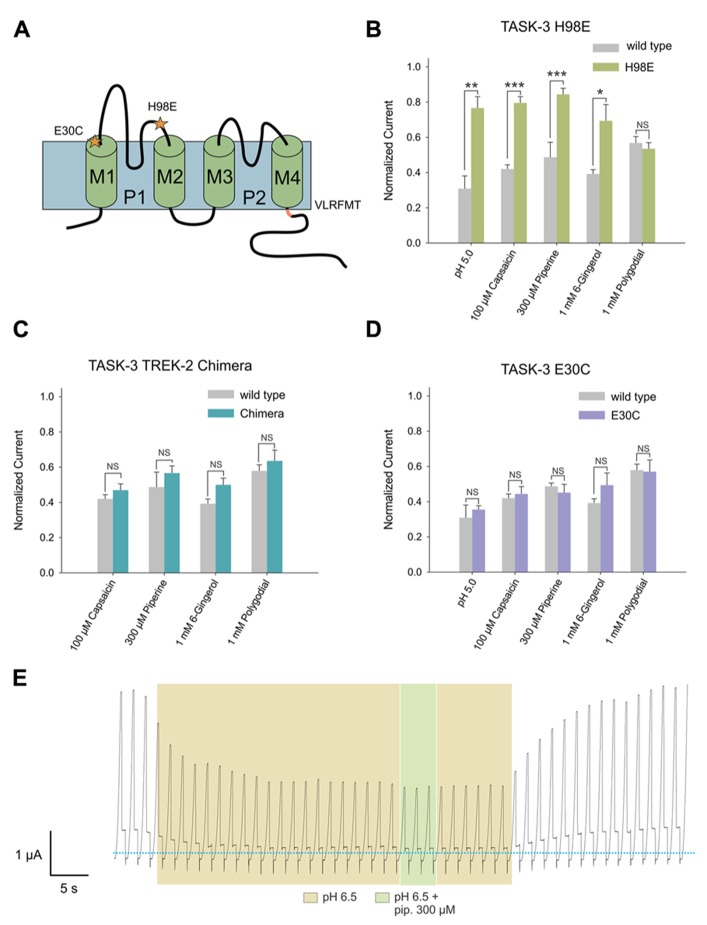
**(A)** Human TASK-3 membrane topology diagram. Four transmembrane segments (M1 to M4) and two pore regions (P1 and P2) are shown. Mutated amino acids are indicated either with a star or as the replaced sequence. **(B)** Comparative effects of pH 5.0, 100 μM capsaicin, 300 μM piperine, 1 mM 6-gingerol and 1 mM polygodial on wild-type human TASK-3 (gray bars) and the H98E mutant (green bars). N of 4–6 oocytes. *P < 0.05, **P < 0.01, ***P < 0.001. **(C)** Comparative effects of the four pungent substances under evaluation in wild-type hTASK-3 (gray bars) vs. TREK-2 chimera (light blue bars). N of 4-5 oocytes. **(D)** Comparative effects of pH 5.0, 100 μM capsaicin, 300 μM piperine, 1 mM 6-gingerol and 1 mM polygodial on wild-type human TASK-3 (gray bars) and the E30C mutant (purple bars). N of 4–6 oocytes. (**E)** Representative voltage clamp recordings of a *Xenopus laevis* oocyte expressing TASK-3 WT exposed to a pH of 6.5 and then to 300 μM piperine.

After evaluating the effect of these pungent substances on K_2P_ channels, we investigated which amino acids could be involved in this interaction. To address this question, we constructed mutants for TASK-3, in which we targeted amino acids necessary for the interaction with well-described agonists or antagonists (**Figure 6A**).

**FIGURE 7:**
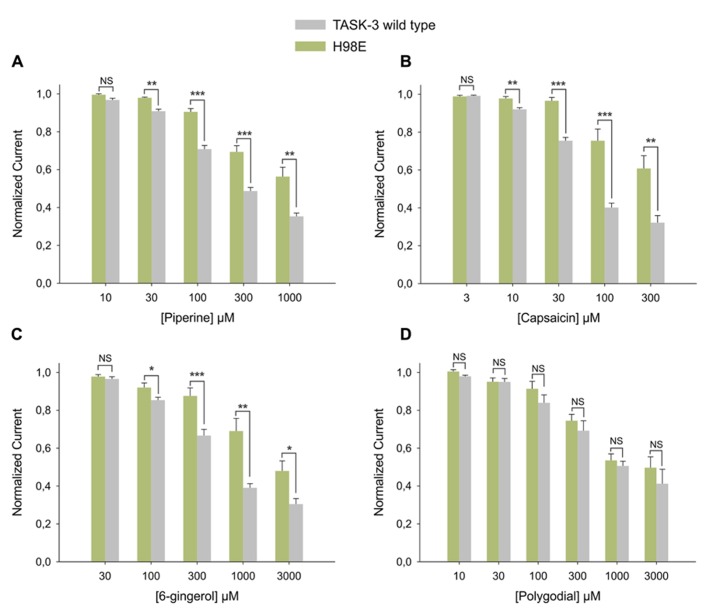
**Dose response relationships for the inhibition induced by pungent substances on TASK-3 WT (gray bars) vs. H98E (green bars) for (A)** piperine, N of 5–12 oocytes; **(B)** capsaicin, N of 5–10 oocytes; **(C)** 6-gingerol, N of 3–19 oocytes and **(D)** polygodial N of 3–9 oocytes. *P < 0.05, **P < 0.01, ***P < 0.001.

First, the histidine at position 98, a key amino acid for the inhibition induced by protons (Rajan et al., 2000; Lopes et al., 2001), was replaced by glutamic acid. The H98E mutant showed significantly less inhibition by 100 μM capsaicin, 300 μM piperine and 1 mM 6-gingerol; 1 mM polygodial, however, had the same effect as on wild-type (**Figure 6B**). Piperine, capsaicin, and 6-gingerol, but not polygodial, induced a significantly decreased inhibitory effect at several other concentrations in the H98E mutant when compared with the wild type (**Figures 7A–D**). In view of this, we investigated whether simultaneous application of piperine and a slightly acidic pH could lead to an increased inhibition of TASK-3 WT. At pH 6.5, which markedly decreased the amount of current carried by TASK-3 but did not abolish it completely, no further inhibition could be observed after the application of 300 μM piperine (**Figure 6E**). This was also the case for TASK-1, in which 300 μM piperine at pH 6.5 induced no further inhibition (not shown). We then constructed a chimera by replacing the VLRFLT_243-248_ sequence of TASK-3, required for volatile anesthetics to induce their activating effect on this channel (Talley and Bayliss, 2002), by the WLRVLS_243__-248_ sequence of human TREK-2. However, this chimera presented no significant difference compared to the wild type in response to any of the four substances (**Figure 6C**). Additionally, in order to investigate if these pungent substances bind preferentially to a closed state of the channel, as it is the case for Zn^2+^, we constructed an E30C mutant, which is reported to induce a closed state in TASK-3 (Veale et al., 2005; Mathie et al., 2010). This mutation, however, produced no significant change in the efficacy of the inhibition induced by any of the four substances at the tested concentrations (**Figure 6D**).

## DISCUSSION

In the present work, we have expanded the pharmacological knowledge about K_2P_ channels. We have shown that the background K^+^ current carried by the human K_2P_ channels TASK-1, TASK-3, and TRESK can be inhibited by piperine, the pungent principle of black pepper, whose mechanism of action has been thought to reside only on its effect on TRPV1. Interestingly this substance presents an IC_50_ on TASK-1 that is similar to its reported EC_50_ on TRPV1-37.9 ± 1.9 μM when evaluated on transfected HEK293 cells (McNamara et al., 2005). The other 3 pungent substances investigated, namely capsaicin, 6-gingerol, and polygodial, also exerted an inhibitory effect, although at concentrations that were almost an order of magnitude higher than their EC_50_ values on TRP channels (Caterina et al., 1997; McNamara et al., 2005; Iwasaki et al., 2006).

Our results call for further studies in order to determine whether K_2P_ channels also play physiological roles in the perception of piperine and other pungent substances. Thus, to investigate the effect of pungent substances on trigeminal ganglia neurons incubated with TRP blockers would be a plausible next step. Moreover, experimental studies are needed to answer the question as to whether these three K_2P_ channels co-express with voltage gated sodium channels, such as Na_v_1.8, which would react to such a depolarizing event, or with TRP channels, whose activating effect would be facilitated by the simultaneous inhibition of K_2P_ channels. It is also worth noticing that the existence of additional players in the perception of piperine and capsaicin, is suggested by findings such as the stimulus-induced recovery (Green, 1998), and since K_2P_ channels do not change the magnitude of their response to any of the tested pungent substances after repeated applications, they deserve to be further studied in regard to this stimulus-induced recovery.

Our mutant studies showed that, when His98 is mutated to Glu, the TASK-3 responses to piperine, capsaicin and 6-gingerol are still present, but significantly reduced at most of the tested concentrations. Interestingly, this amino acid is thought to be the main proton sensor in TASK-1 and TASK-3 (Rajan et al., 2000; Lopes et al., 2001), and, when mutated, leads to a marked change in proton sensitivity (Rajan et al., 2000). For TASK-1 the postulated mechanism involves a water molecule behind the selectivity filter, which interacts with His98 and other 3 amino acids in order to stabilize the backbone of the selectivity filter. According to this model, when His98 is protonated, it rotates up and consequently an electropositive barrier to K^+^ at the outer mouth of the channel is created (Stansfeld et al., 2008). The large molecular size of capsaicin, piperine, and 6-gingerol makes it difficult to suggest that they might inhibit TASK-3 by means of the same mechanism. However, the fact that the same concentrations of the above mentioned substances induced a significantly greater inhibition in WT in comparison to the His98 mutant, strongly suggest that this amino acid might play a role in the inhibition induced by these phytochemicals.

## Conflict of Interest Statement

The authors declare that the research was conducted in the absence of any commercial or financial relationships that could be construed as a potential conflict of interest.

## AUTHOR CONTRIBUTIONS

Conceived and designed the experiments: Hanns Hatt, Thomas Hofmann, Guenter Gisselmann, Leopoldo R. Beltrán, Corinna Dawid, Madeline Beltrán. Performed the experiments: Leopoldo R. Beltrán, Corinna Dawid, Madeline Beltrán, Katharina Degenhardt, Klaus Mathie. Analyzed the data: Leopoldo R. Beltrán, Madeline Beltrán. Wrote the paper: Leopoldo R. Beltrán, Corinna Dawid, Madeline Beltrán. Leopoldo R. Beltrán, Corinna Dawid, Madeline Beltrán contributed equally to the present work.
